# Editorial: Eudaimonia and Music Learning

**DOI:** 10.3389/fpsyg.2021.735393

**Published:** 2021-08-18

**Authors:** Gareth Dylan Smith, Marissa Silverman, Dylan van der Schyff

**Affiliations:** ^1^Music Education Department, Boston University, Boston, MA, United States; ^2^Music Education Department, Montclair State University, Montclair, NJ, United States; ^3^Conservatorium of Music, The University of Melbourne, Parkville, VIC, Australia

**Keywords:** editorial, music, learning, eudaimonia, flourishing

This special topic came about in response to a recent surge in interest and scholarship around the combined themes of eudaimonia and music learning. In a way, musicians, music teachers, and scholars of music learning have always been writing about eudaimonia and music learning; they just have not always phrased it as such. *Music learning* is an obvious and necessary topic of discussion for musicians, teachers, and community music practitioners. It is a very diverse field, including the broad range of perspectives and practices that fall under the rubric of “music education” (a term understood and interpreted as variously as the international contexts in which it is used) and much more. *Eudaimonia* is probably less familiar to some, since writing on this term is far sparser. As contributors to this special topic have noted elsewhere, eudaimonia is a concept that began its life with Plato and Aristotle, and has been interpreted according to prevailing paradigms in Western secular and religious thought ever since (Boyce-Tillman, 2020; Silverman, 2020a; Smith and Silverman, 2020; van der Schyff, 2020). Eudaimonism—the ethos of or orientation to eudaimonia—is an ethical frame that focuses on human flourishing or thriving. It is a concept that runs through literature on positive psychology (Seligman, 2011), connected with research happiness, with fulfillment-based conceptions eudaimonia often differentiated from the more immediate gratification and pleasures of hedonic happiness (Deci and Ryan, 2008). Writing on eudaimonia tends to focus either more on individual purpose and self-fulfillment (Norton, 1976; Waterman, 1992; Smith, 2016) or more on collective well-being and the responsibilities of learners, educators, and facilitators in that regard; music education scholars have tended to write more on the latter (Elliott and Silverman, 2014, 2015; Elliott, 2020).

While in a way, this special topic broaches little that is startlingly new, it curates a space for papers by like-minded thinkers and writers who find the eudaimonic frame to be helpful. The content of this special topic is far from comprehensive in its purview, which as editors and scholars in this domain we find exciting, hopeful that readers might find this an area generative of further thinking themselves.

In June Boyce-Tillman's paper—“Heart's ease: Eudaimonia, musicking and the pandemic and its implications for music education”—the author discusses themes in the eudaimonia literature, comprising ethical behavior, a sense of meaning and purpose, contemplation, relationship with spirits of the ancestors and celestial beings, and relationships of mutuality and respect. She then discusses, in the context of the COVID-19 pandemic, how music educators have adopted and enacted these values, before suggesting ways in which music teaching and learning can continue to grow.

In their paper titled “Flourishing in resonance: Joint resilience building through music and motion,” Luc Nijs and Georgia Nicolaou situate their work by discussing connections between conceptions of resilience and eudaimonia. They go on to describe how musical activities founded in movement practices can help young people to develop and experience eudaimonic values that include self-awareness, confidence and self-esteem, personal autonomy, connection, belonging, and bonding.

What are the aims, purposes, and perceptions of older musicians who participate in blues jam sessions? In “Jammin' the Blues: Experiencing the ‘Good Life,” Debrot examines and analyzes how eudaimonic well-being results from life-long musicing. Moreover, because of the participatory nature of the jam sessions, and because participatory musicing yields agency and collective self-other direction, adult music makers at The Tavern on the Hill expanded their social, cultural, and musical identities in profound and meaningful ways.

Meanwhile, Hendricks et al., in “Caring For, About, and With: Exploring Musical Meaningfulness Among Suzuki Students and Parents,” examined parent-child shared music learning experiences through Suzuki training, and how such music making and sharing might assist in the development of empathy, connectivity, and relationality between parents and their children. The authors note, “music learning, human connections, and shared values work together to reinforce musical meaningfulness, potentially creating a self-perpetuating eudaimonic circle.”

In “A Space for Collaborative Creativity: How Collective Improvising Shapes a Sense of Belonging,” Verneert et al., examine the eudaimonic potential and potencies of adult music makers, namely during collective musical improvisational experiences. The community under examination, here, was homeless adults as well as individuals with emotional, psychological, and cognitive challenges. For these individuals engaged in non-formal, community musicing, the researchers found results such as a sense of belonging, “flow” (or optimal experience), meaningfulness (see also Silverman, 2020b), as well as accomplishment and satisfaction.

Smith's contribution—“Music education for surviving and thriving: Cultivating children's wonder, senses, emotional well-being, and wild nature as a means to discover and fulfill their life's purpose”—develops connections between the eudaimonic perspective and eco-literate music pedagogy. Smith draws on insights from ecological philosophy, soundscape studies, and more to show how these mutually supporting orientations can help to dismantle the “logic of domination” that pervades modern life. She then discusses how musical praxis informed by this critical shift in thinking can “encourage children (and all people) to maintain their sense of wonder in nature, to fully develop their sensory capacities, to support their psychospiritual well-being, and to foster soulcentric maturation.”

Walzer's paper, “Fostering trauma-informed and eudaimonic pedagogy in music education,” takes a look through several theoretical lenses at what he describes as a praxis of care in music education. Beginning in the context of music making and learning during the COVID-19 pandemic, Walzer urges music educators to consider a eudaimonic pedagogy, based in humility, confidence, and a nurturing, loving approach to music teaching and learning.

## Author Contributions

All authors listed have made a substantial, direct and intellectual contribution to the work, and approved it for publication.

## Conflict of Interest

The authors declare that the research was conducted in the absence of any commercial or financial relationships that could be construed as a potential conflict of interest.

## Publisher's Note

All claims expressed in this article are solely those of the authors and do not necessarily represent those of their affiliated organizations, or those of the publisher, the editors and the reviewers. Any product that may be evaluated in this article, or claim that may be made by its manufacturer, is not guaranteed or endorsed by the publisher.
